# Interstitial fluid osmolarity modulates the action of differential tissue surface tension in progenitor cell segregation during gastrulation

**DOI:** 10.1242/dev.144964

**Published:** 2017-05-15

**Authors:** S. F. Gabriel Krens, Jim H. Veldhuis, Vanessa Barone, Daniel Čapek, Jean-Léon Maître, G. Wayne Brodland, Carl-Philipp Heisenberg

**Affiliations:** 1 Institute of Science and Technology Austria, 3400 Klosterneuburg, Austria; 2 Department of Civil and Environmental Engineering, University of Waterloo, Waterloo N2L 3G1, Canada

**Keywords:** Tissue surface tension, Cell internalization, Gastrulation, Zebrafish

## Abstract

The segregation of different cell types into distinct tissues is a fundamental process in metazoan development. Differences in cell adhesion and cortex tension are commonly thought to drive cell sorting by regulating tissue surface tension (TST). However, the role that differential TST plays in cell segregation within the developing embryo is as yet unclear. Here, we have analyzed the role of differential TST for germ layer progenitor cell segregation during zebrafish gastrulation. Contrary to previous observations that differential TST drives germ layer progenitor cell segregation *in vitro*, we show that germ layers display indistinguishable TST within the gastrulating embryo, arguing against differential TST driving germ layer progenitor cell segregation *in vivo*. We further show that the osmolarity of the interstitial fluid (IF) is an important factor that influences germ layer TST *in vivo*, and that lower osmolarity of the IF compared with standard cell culture medium can explain why germ layers display differential TST in culture but not *in vivo*. Finally, we show that directed migration of mesendoderm progenitors is required for germ layer progenitor cell segregation and germ layer formation.

## Abstract (precis)

**Highlighted Article:** Segregation of the germ layer progenitors in the zebrafish gastrula is driven by directed cell migration, rather than differential tissue surface tension.

## INTRODUCTION

During gastrulation, the germ layer progenitor cell types – ectoderm, mesoderm and endoderm – segregate into distinct germ layers with ectoderm positioned on the outside of the embryo and mesoderm and endoderm on its inside (Stern, 2004). In zebrafish embryos, progenitor cell segregation is initiated by progenitor cells that have been induced to become mesoderm or endoderm internalizing at the germ ring margin, thereby forming the mesendoderm (hypoblast) below the non-internalizing ectoderm (epiblast; Montero et al., 2005; Rohde and Heisenberg, 2007; Solnica-Krezel and Sepich, 2011; Warga and Kimmel, 1990).

The molecular, cellular and biophysical mechanisms that underlie cell segregation and tissue self-organization have been studied for decades (Borghi and Nelson, 2009). Differences in cell adhesion and cortical tension, which together determine tissue surface tension (TST), are generally thought to constitute crucial determinants that drive cell sorting and tissue layering in development (Foty and Steinberg, 2013; Krens and Heisenberg, 2011). In zebrafish and *Xenopus* gastrulation, differential TST between the forming germ layers has been postulated to trigger progenitor cell segregation and germ layer positioning (Krieg et al., 2008; Maître et al., 2012; Schötz et al., 2008). However, evidence that supports this view has so far nearly exclusively come from experiments performed on cells and tissues in culture. Moreover, studies in *Xenopus* embryos have suggested that cadherin-dependent differential TST causes cell sorting *in vitro*, but not in the embryo (Ninomiya et al., 2012). The main difficulty in determining the contribution of differential TST to cell sorting *in vivo* has been the lack of techniques for determining TST within the physiological environment where these processes naturally occur.

Here, we introduce CellFIT-3D, a 3D force inference method (Brodland et al., 2010, 2014) that allows us to analyze TST within the zebrafish gastrula. Combining this tool with live cell imaging and genetic perturbation, we provide evidence that directed cell migration rather than differential TST drives progenitor cell segregation *in vivo*, and that osmolarity of the surrounding fluid is an important factor influencing germ layer TST.

## RESULTS

**Fig. 1.**
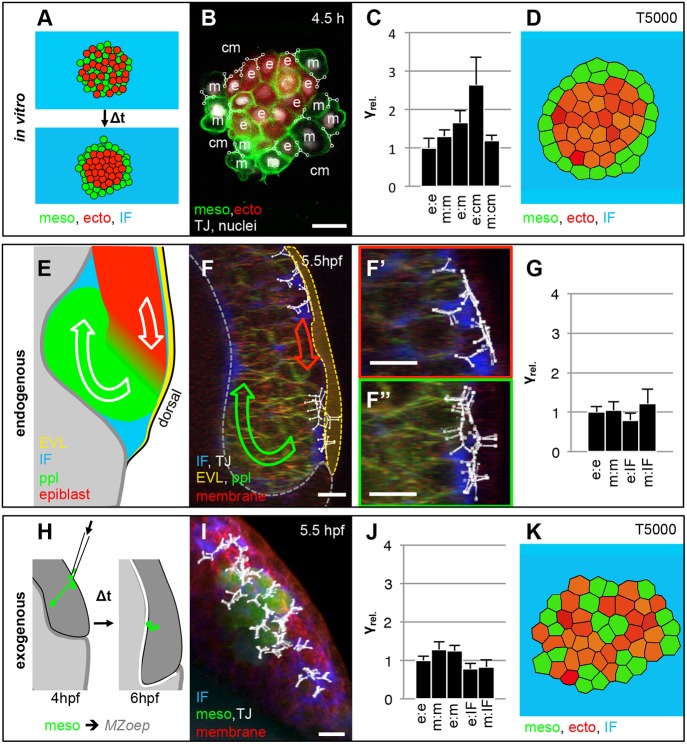
**Relative interfacial tension distribution during cell segregation *in vitro* and *in vivo*.** (A) Schematic illustration of the starting and end configurations for a typical heterotypical progenitor cell-sorting assay *in vitro*. (B) Single confocal image plane of a heterotypical aggregate consisting of ectoderm (ecto) and mesoderm (meso) progenitor cells expressing histone2A-mCherry in the nucleus (white) and Lyn-Venus at the plasma membrane (green) in all cells after 4.5 h in culture. Ectoderm progenitor cells were additionally labeled with cytoplasmic dextran-Alexa647 (red). Representative cell triple interfacial junctions (TJ, white) for cell-to-cell and cell-to-medium interfaces, as part of the CellFIT-3D based tensions analysis, were overlaid in the image as triple nodes in white with the different interfaces denoted as e (ectoderm), m (mesoderm) and cm (culture medium). Scale bar: 20 μm. For more details of the CellFIT-3D, see the supplementary Materials and Methods. (C) Relative interfacial tension distributions (γ_rel._) obtained by CellFIT-3D for all interface types present during *in vitro* cell sorting at 4.5 h in culture. Error bars show standard deviations. (D) Stable configurations of a finite element simulation of heterotypical progenitor cell sorting after 5000 computational iterations, using the CellFIT-3D obtained interfacial tensions shown in C with γ_e-e_=1.00, γ_m-m_=1.31, γ_e-m_=1.66, γ_e-cm_=2.65 and γ_m-cm_=1.20. (E) Schematic illustration of mesoderm internalization in a lateral view through the dorsal germ ring margin at the onset of gastrulation. (F) 3D-rendered image of a Tg*(gsc:eGFP)* embryo at the onset of internalization (5.5 hpf) with ppl progenitor cells expressing eGFP (green), all cells expressing membrane-labeled Lyn-TagBFP (red), and the IF marked by dextran-rhodamine (blue). The image is overlaid with annotated triple junctions (TJ, white). The green and red arrows indicate global movement directions of mesoderm and ectoderm progenitor cells, respectively. The yellow dotted line demarcates the EVL. Scale bar: 20 μm. (F′,F″) Higher magnification views of the regions with ectoderm cells (F′, red) and ppl progenitor cells expressing eGFP (F″, green) from the image in F. Scale bars: 20 μm. (G) Relative interfacial tension distributions (γ_rel._) obtained by CellFIT-3D for all interface types present during gastrulation *in vivo* at 5.5 h with e (ectoderm), m (mesoderm) and IF (interstitial fluid). Error bars show standard deviations. (H) Schematic illustration of a typical transplanted mesoderm cell internalization experiment. (I) 3D-rendered image of Tg*(βActin:Ras-eGFP)* mesoderm cells (green) transplanted in a Lyn-TagBFP membrane-labeled (red) expressing Tg*(dharma:eGFP);MZoep* embryo at the onset of internalization (5.5 hpf) with the IF marked by dextran-rhodamine (blue) and overlaid with annotated triple junctions (TJ, white). Scale bar: 20 μm. (J) Relative interfacial tensions obtained by CellFIT-3D at the onset of mesoderm internalization with e (ectoderm), m (mesoderm) and IF (interstitial fluid). Error bars are standard deviations. (K) Stable configurations of a finite element simulation of heterotypical progenitor cell sorting after 5000 computational iterations, using the CellFIT-3D obtained interfacial tensions shown in J with γ_e-e_=1.00, γ_m-m_=1.28, γ_e-m_=1.25, γ_e-IF_=0.78 and γ_m-IF_=0.83.

To analyze the potential contribution of TST to progenitor cell segregation during gastrulation, we developed a new version of video force microscopy, CellFIT-3D, that is capable of analyzing interfacial tensions in cells from three-dimensional (3D) confocal stacks (Brodland et al., 2010, 2014). First, we validated our CellFIT-3D method by analyzing TST during cell segregation in heterotypic aggregates of ectoderm and mesoderm progenitor cells *in vitro* (Fig. 1A; Movie 1), previously shown to be driven by differential TST (Krieg et al., 2008; Maître et al., 2012; Schötz et al., 2008). For our analysis, we considered five different types of interfaces: two homotypic cell-cell interfaces (ectoderm-ectoderm, mesoderm-mesoderm), one heterotypic cell-cell interface (ectoderm-mesoderm) and two cell-fluid interfaces (ectoderm-medium, mesoderm-medium) (Fig. 1B). Consistent with biophysical measurements (Krieg et al., 2008; Maître et al., 2012; Schötz et al., 2008), our CellFIT-3D analysis revealed a higher ratio of cell-medium to homotypic cell-cell interfacial tensions in ectoderm compared with mesoderm cells (Fig. 1C), indicative of ectoderm displaying higher TST than mesoderm. This confirms previous findings of stronger actin and myosin II localization at cell-medium interfaces in ectoderm compared with mesoderm progenitors (Krieg et al., 2008; Maître et al., 2012; Fig. S1), and is consistent with the assumption that differential TST between ectoderm and mesoderm drives progenitor cell segregation *in vitro* (Schötz et al., 2008). It further supports the notion that CellFIT-3D is a reliable method with which to determine germ layer TST and analyze the specific contribution of differential TST to germ layer progenitor cell sorting.

For analyzing TST between ectoderm and mesoderm cells during cell segregation *in vivo*, we applied our CellFIT-3D method to confocal time-lapse movies of anterior axial mesendoderm (prechordal plate, ppl) cell internalization within the dorsal germ ring margin at the onset of gastrulation [5-6 h post fertilization (hpf) Fig. 1E; Movie 2]. We chose to analyze ppl progenitor cells, because they are easy to identify in the developing embryo and show features common to mesendoderm progenitor cell internalization during gastrulation (Montero et al., 2005). As in our *in vitro* analysis, we considered the ratio of progenitor cell-fluid (interstitial fluid; IF) to homotypic cell-cell interfacial tensions as a read-out for germ layer TST (Maître et al., 2012). Surprisingly, upon analyzing more than 450 manually digitized angle sets of 119 cell contacts using CellFIT-3D (Fig. 1F,F′,F″), we found that, different from the situation in culture (Krieg et al., 2008; Maître et al., 2012; Schötz et al., 2008), TST of ectoderm and mesoderm were largely indistinguishable *in vivo* (Fig. 1G). To further validate this observation, we also analyzed TST during internalization of ppl progenitors that were transplanted directly below the surface of the dorsal germ ring of pre-gastrula stage (40% epiboly; 5 hpf) MZ*oep* mutant embryos lacking endogenous mesendoderm cells (Fig. 1H; Movie 3; Gritsman et al., 1999). In contrast to the situation of endogenous ppl cell internalization, where unambiguously locating heterotypic interfaces between mesoderm and ectoderm progenitors was impossible, this transplantation assay also allowed us to identify clearly and analyze these heterotypic interfaces. Similar to the endogenous situation, we found indistinguishable TST between ectoderm and mesoderm upon analysis of about 200 angle sets obtained from 60 cell contacts (Fig. 1I,J). Together, these analyses suggest that, unlike the situation *in vitro* (Krieg et al., 2008; Maître et al., 2012; Schötz et al., 2008), ectoderm and mesoderm display indistinguishable TST during mesoderm internalization at the onset of gastrulation. It further points to the possibility that while differential TST is sufficient to drive progenitor cell segregation *in vitro*, it might not have such a function within the embryo.

To further test this possibility, we asked to what extent the relative interfacial tension values obtained by CellFIT-3D during progenitor cell segregation *in vitro* versus *in vivo* can trigger progenitor cell segregation *in silico*. To this end, we performed simulations of TST-driven cell segregation using Finite Element (FE)-based forward modeling (Brodland, 2004; Brodland et al., 2007). We started our simulations with a configuration of randomly intermixed ectoderm and mesoderm cells forming a coherent cluster that is surrounded by a liquid medium, equivalent to the actual situation of progenitor cell sorting *in vitro* (Krieg et al., 2008; Maître et al., 2012; Schötz et al., 2008). When using the relative interfacial tension values obtained from the *in vitro* cell segregation experiments, ectoderm and mesoderm cells were efficiently segregating into a configuration where mesoderm surrounded ectoderm (Fig. 1D; Movie 4, left). By contrast, when the relative interfacial tension values found *in vivo* were used, no progenitor cell segregation was observed (Fig. 1K; Movie 4, right). These findings support our assumption that differential TST is sufficient to drive progenitor cell segregation *in vitro* but not *in vivo*.

**Fig. 2.**
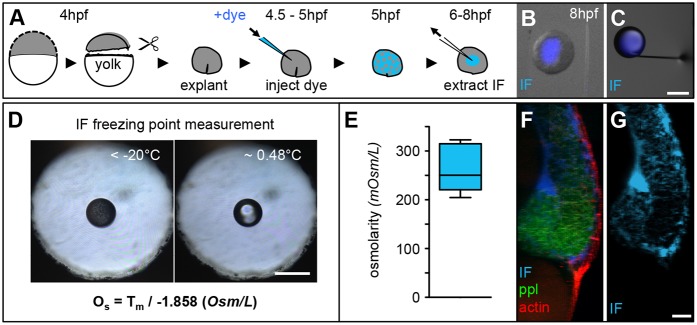
**Measurements of interstitial fluid osmolarity.** (A) Schematic illustration of animal cap explant preparation, and IF labelling and extraction procedures. (B,C) Overlaid images of an animal cap explant (gray scale) with IF-filled lumen (blue; B) and an extracted IF droplet (blue) at the tip of a glass capillary used for IF extraction (C). Scale bar: 200 μm. (D) Images of a droplet of IF floating in immersion oil placed within the sample-holder hole of a nanoliter osmometer in a frozen state (left) and directly after melting (right). The displayed formula was used to calculate the IF osmolarity, with O_s_ denoting osmolarity (Osm/L) and T_m_ being the temperature of freeze point depression. Scale bar: 50 μm. (E) Box and whisker plot of the measured IF osmolarity (*n*=8 measurements). Black line indicates the median value; whiskers show the spread of the data. (F,G) 3D renderings of a lateral fluorescence image of the dorsal germ ring in a Tg*(-4gsc:eGFP-Hsa.HRAS)* embryo at the onset of gastrulation (6 hpf), expressing GFP in internalizing ppl progenitors (green), utrophin-mCherry to label the actin cell cortex of all cells (red) and dextran-Cascade Blue to label the IF (blue; F). (G) The same image showing dextran-Cascade Blue labeling of IF only. Scale bar: 20 μm.

Our analysis raises two main questions: (1) why are cell interfacial tensions different in the embryo compared to the situation in culture; and (2) what mechanism(s) – if not differential TST – drive progenitor cell segregation *in vivo*? In addressing the first question, we reasoned that differences between the physiological environment *in vivo* and cell culture conditions *in vitro* might be responsible. To identify those differences, we searched for factors that might vary between the situation *in vivo* and *in vitro*, and have the potential to affect cell interfacial tensions. There is increasing evidence that osmolarity of the surrounding medium plays an important role in determining hydrostatic cell pressure and, consequently, cell cortex tension, a crucial cell property that influences cell interfacial tensions (Salbreux et al., 2012; Stewart et al., 2011). We thus speculated that IF *in vivo* might have a different osmolarity than cell culture medium used *in vitro*, and that this difference might be responsible for the observed discrepancy between TST *in vivo* versus *in vitro*. To address this hypothesis, we first sought to determine osmolarity of the IF *in vivo* at the onset of gastrulation. To this end, we made use of a nanoliter osmometer (Otago osmometers; Braslavsky and Drori, 2013) that allows measuring the osmolarity of small fluid quantities (≈10 nL). As the total amount of IF per embryo is very small (≈15 nL) and distributed between progenitor cells throughout the gastrula (Fig. 2F,G), we were unable to extract sufficient amounts of IF directly from embryos. Instead, we made use of blastoderm explants (animal caps) excised from sphere-stage embryos (4 hpf; Krens et al., 2011), which formed a clearly recognizable accumulation of IF at the explant interior when kept in culture for ∼2-3 h (Fig. 2A). We then extracted IF from multiple of those explants by micropipette aspiration (Fig. 2B,C) and analyzed the osmolarity of the extracted IF using our nanoliter osmometer (Fig. 2D). Strikingly, we found that the osmolarity of the IF was considerably lower (250.3±47.4 mOsm/L) than the osmolarity of the cell culture medium typically used to study progenitor cell sorting *in vitro* (∼300 mOsm/L; Fig. 2E).

**Fig. 3.**
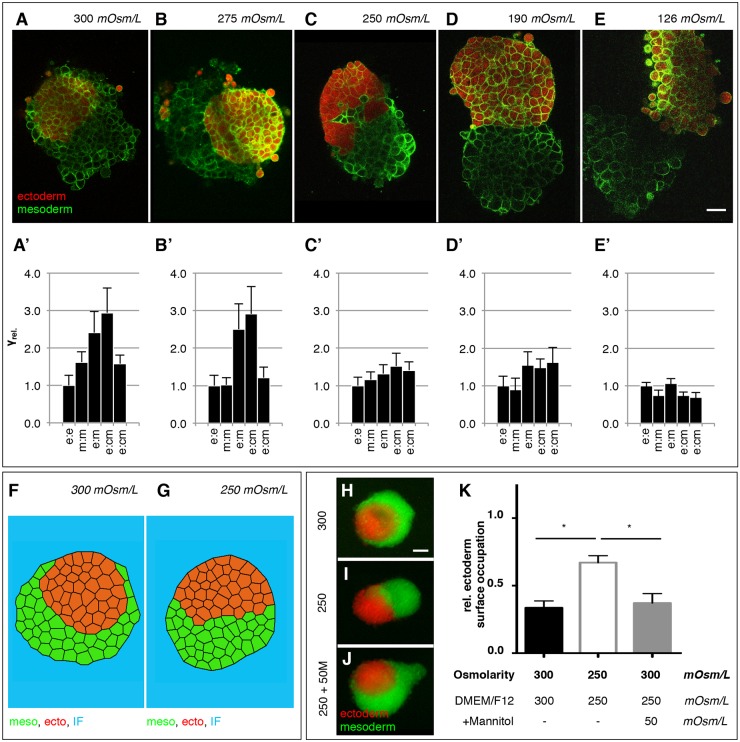
**Modulation of progenitor cell interfacial tensions by medium osmolarity.** (A-E) Representative single plane confocal images of tissue aggregates consisting of ectoderm or mesoderm progenitor cells expressing Lyn-Venus at the plasma membrane (green) of all cells and cultured for 5 h in the presence of medium with 300 (A), 275 (B), 250 (C), 190 (D) or 126 (E) mOsm/L osmolarity. Ectoderm aggregates were additionally labeled with cytoplasmic dextran-Alexa648 (red) (see also Movie 3). Scale bar: 50 μm. (A′-E′) Relative interfacial tensions (γ_rel._) obtained by 3D-CellFIT for enveloping tissues cultured for 5 h in the presence of medium with 300 (A′), 275 (B′), 250 (C′), 190 (D), 126 (D′) or 250 (E′) mOsm/L osmolarity with e (ectoderm), m (mesoderm) and cm (culture medium). Error bars indicate standard deviations. (F,G) Stable configurations of finite element simulations of tissue envelopment (10,000 simulation iterations) in heterotypic tissue aggregates consisting of ectoderm (red) or mesoderm (green) cells surrounded by culture medium (blue), using 3D-CellFIT-obtained tension distributions shown in A (300 mOsm/L) with γ_e-e_=1.00, γ_m-m_=1.62, γ_e-m_=2.41, γ_e-cm_=2.94, γ_m-cm_=1.58; and in C (250 mOsm/L) with γ_e-e_=1.00, γ_m-m_=1.16, γ_e-m_=1.32, γ_e-cm_=1.52, γ_m-cm_=1.41. (H-J) Tissue envelopment of ectoderm (red) and mesoderm (green) progenitor cell aggregates cultured for 5 h in the presence of ∼300 mOsm/L culture medium (*n*=20 engulfment assays; H), ∼250 mOsm/L osmolarity culture medium (*n*=24 engulfment assays; I) or culture medium containing mannitol to restore osmolarity from 250 mOsm/L to 300 mOsm/L (250+50 M; *n*=21 engulfment assays, J). (K) Degree of envelopment was quantified by calculating the relative ectoderm surface occupation taking the heterotypical cell aggregate size into account. Error bars are standard deviations; *P<0.05.

To determine whether this difference in osmolarity between IF and culture medium might be responsible for the observed differences in germ layer TST between the situations *in vivo* (IF) versus *in vitro* (cell culture medium), we used our 3D-CellFIT method to analyze TST of germ layer explants cultured in the presence of culture medium with osmolarity ranging from 126 to 300 mOsm/L. Consistent with previous observations (Krieg et al., 2008; Schötz et al., 2008), we found that in 300 mOsm/L culture medium*,* ectoderm explants displayed higher TST than mesoderm (Fig. 3A′), and that this differences caused mesoderm to envelop ectoderm when placing these explants adjacent to each other (Fig. 3A). By contrast, when explants were cultured in medium with an osmolarity similar or lower to that of the IF (250-126 mOsm/L; Fig. 3C-E), mesoderm and ectoderm explants displayed indistinguishable TST (Fig. 3C′-E′) and, consequently, there was no envelopment observed when these tissues were brought into contact with each other (Fig. 3C-E). Interestingly, ectoderm and mesoderm tissues still displayed differential TST and, consequently, mesoderm enveloped ectoderm in the presence of culture medium with an osmolarity intermediate between the osmolarity of standard culture medium and IF (275 mOsm/L; Fig. 3B,B′). This suggests that medium/IF osmolarity must be at least as low as 250 mOsm/L for differential TST between ectoderm and mesoderm to vanish. This conclusion was further supported by simulations of explant envelopment with the interfacial tension values obtained from our 3D-CellFIT analyses using FE-based forward modeling, producing envelopment behaviors similar to the ones observed in the experiments (Fig. 3F,G; Movies 5 and 6), confirming that osmolarity-induced changes in explant envelopment were indeed due to associated changes in explant TST. Additionally, to test the validity of our 3D-CellFIT based findings on interfacial tension values in the presence of culture medium with different osmolarity, we directly measured cell-medium interfacial tensions (cortical tensions) of individual ectoderm and mesoderm progenitors in the presence of high (300 mOsm/L) versus low (190 mOsm/L) osmolarity culture medium using single cell force spectroscopy (Krieg et al., 2008). Consistent with our 3D-CellFIT data, cell-medium interfacial tension was higher in ectoderm compared with mesoderm progenitors in the presence of culture medium with 300 mOsm/L, whereas no such difference was detectable anymore when medium osmolarity was lowered to 190 mOsm/L (Fig. S1). Collectively, these findings support our initial assumption that osmolarity affects TST, and that differences in the osmolarity between IF and cell culture medium can explain the discrepancy in the measured TST *in vivo* versus *in vitro*.

Finally, we asked whether the observed effect of culture medium osmolarity on germ layer explant TST and, consequently, their envelopment behavior in culture, was indeed mediated by changes in medium osmolarity rather than alterations in the concentration of specific culture medium ingredients. To this end, we tested whether increasing culture medium osmolarity from 250 to 300 mOsm/L by adding the non-ionic osmolyte mannitol, a sugar frequently used to manipulate culture medium osmolarity (Enyedi et al., 2013), would have the same effect on explant envelopment behavior as observed when placing these explants directly into 300 mOsm/L culture medium. We found that the addition of mannitol to the culture medium induced germ layer explant envelopment to a similar degree to that observed when placing heterotypical explants directly into 300 mOsm/L culture medium (Fig. 3H-K). This suggests that, in our experiments, changes in medium osmolarity, rather than the concentration of specific culture medium ingredients, affected germ layer TST.

**Fig. 4.**
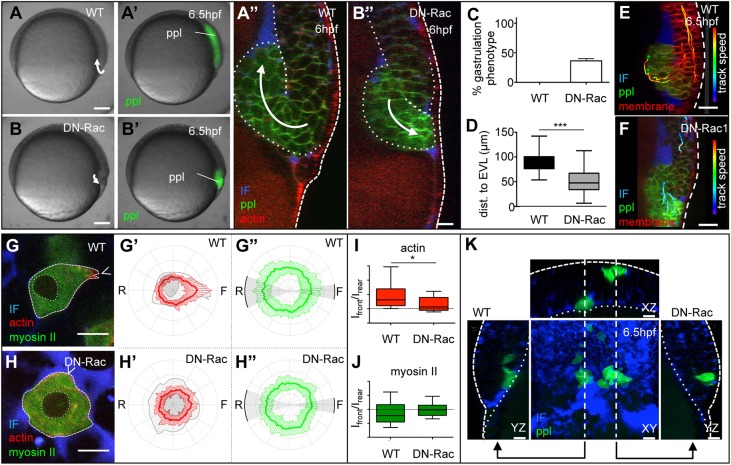
**Mesoderm cell internalization relies on directed mesoderm cell migration*.*** (A,A′,B,B′) Bright-field (A,B) and fluorescence images (A′,B′) of wild-type (A,A′) and DN-Rac expressing (B,B′) Tg*(-4gsc:eGFP-Hsa.HRAS)* embryos, expressing GFP in internalizing ppl progenitors (green) at the onset of gastrulation (5.5 hpf). Scale bars: 100 μm. (A″,B″) Orthogonal view of a confocal image stack of a wild-type (A″) and DN-Rac-expressing (B″) Tg*(-4gsc:eGFP-Hsa.HRAS)*;Tg*(βActin:Utrophin-mCherry)* embryo at the onset of gastrulation (6 hpf), expressing GFP (green) in internalizing ppl progenitors and Utrophin-mCherry (red) marking actin in all cells. Transgenic embryos were also injected with dextran-rhodamine to label the IF (blue). Dashed line delineates the position of the EVL and dotted lines demarcate the deep cell mass. White arrows indicate the direction of mesoderm progenitor cell movement. Scale bar: 20 μm. (C) The fraction of DN-Rac-expressing and wild-type control embryos displaying defective mesoderm cell internalization (*n*_WT_=124 and *n*_DN-Rac_=95 embryos from three independent experiments). Error bars are standard deviations. (D) Box and whisker plot showing the distance of internalized mesoderm progenitors from the EVL in wild-type and DN-Rac-expressing embryos (*n*_WT_ and *n*_DN-Rac_=264 cells from three embryos each; ***P<0.01). Black line indicates the median value; whiskers show the spread of the data. (E,F) Orthogonal views from a confocal image stack of internalizing ppl progenitors within the dorsal germ ring margin of wild-type (E) and DN-Rac-expressing (F) *Tg(-4gsc:eGFP-Hsa.HRAS)* embryo, expressing GFP in ppl progenitors (green) at shield stage (6.5 hpf). Transgenic embryos were also injected with *Lyn-TagBFP m*RNA to outline the membrane of all cells (red) and dextran-rhodamine to label the IF (blue). Cell tracks delineate the movements of exemplary mesoderm (green spheres) and ectoderm cells (red spheres) during internalization (5.7-6.5 hpf). Track speed calibration bar indicates cell migration speeds ranging from 0 to 3.6 μm/min, from blue to red*.* Scale bars: 20 μm. Dashed line delineates the position of the EVL. (G,H) Single plane confocal image of an exemplary transplanted Tg*(βActin:myl12l-eGFP)*;Tg*(βActin:Utrophin-mCherry)* wild-type (G) and DN-Rac-expressing (H) ppl progenitor cell *in vivo* during internalization. The surrounding IF was labeled with dextran-Cascade Blue (blue). Scale bars: 10 μm. Arrowhead indicates the cell front. (G′,G″,H′,H″) Normalized fluorescence intensity values on the periphery of wild-type (G′,G″) and DN-Rac-expressing (H′,H″) ppl cells in Tg*(βActin:myl12l-eGFP)*;Tg*(βActin:Utrophin-mCherry)* embryos at shield stage (6 hpf) shown as polar plots for actin (Utrophin-mCherry; G′,H′) and myosin II (myl12l-eGFP; G″,H″) with the front and rear areas annotated. Standard deviations are indicated as lines perpendicular to the mean. *n*=18 cells (5 embryos) for wild type; *n*=16 cells (4 embryos) for DN-Rac. (I,J) Box and whisker plots of actin (I) and myosin II (J) intensity ratios of the front versus the rear in wild-type and DN-Rac-expressing ppl cells. Black line indicates the median value; whiskers show the spread of the data; dashed line indicate a value of 1 (equal intensity front versus rear); *P<0.05. (K) 3D rendered image planes [XY, dorsal view with animal pole towards the top; XZ, transverse view with outside (EVL) towards the top; YZ, lateral view with animal pole towards the top] of wild-type (left) and DN-Rac-expressing (right) Tg*(-4gsc:eGFP-Hsa.HRAS)* donor cells (green) transplanted adjacent to each other into the dorsal germ ring margin of a wild-type host embryo at shield stage (6 hpf). Host embryos were injected with dextran-rhodamine to label the IF (blue). Straight dashed lines in the middle panel indicate the image planes of the neighboring panels. Dotted lines outline the deep cell mass. ppl, prechordal plate progenitor cells; EVL, enveloping layer; IF, interstitial fluid. Scale bars: 20 μm.

Our findings so far suggest that germ layer progenitor cells do not display differential TST *in vivo*, and, consequently, that differential TST is unlikely to drive progenitor cell segregation during gastrulation. To investigate which mechanism(s) – if not differential TST – then drive progenitor cell segregation within the embryo, we performed multi-photon time-lapse imaging of endogenous ppl progenitors internalizing at the dorsal germ ring at the onset of gastrulation (5-6 hpf; Fig. 4A-A″,E; Movie 7). We found that individual ppl progenitor cells marked by their expression of GFP in Tg*(gsc:GFP)* embryos moved from the outside to the inside of the germ ring margin, a behavior characteristic of progenitor cell ingression (Montero et al., 2005). Moreover, internalizing ppl progenitor cells, but not non-internalizing ectoderm cells, displayed features typically associated with migrating cells, such as preferentially localizing actin to their protrusive front-end (Fig. 4G,G′; Fig. S2,3). Myosin II localization, by contrast, did not show any preferential localization to the leading or trailing edges of internalizing ppl cells (Fig. 4G″), and exposing cultured ppl cells to the myosin II inhibitor blebbistatin did not interfere with protrusion formation in these cells (Fig. S3). Together, this points to the possibility that ppl progenitors segregate from non-internalizing ectoderm progenitors by undergoing directed cell migration. To further test this possibility, we asked whether interfering with the migratory capacity of ppl progenitors would disrupt progenitor cell ingression and thus segregation within the germ ring. To interfere with ppl cell migration, we expressed a dominant-negative version of Rac (DN-Rac), previously shown to reduce cell protrusion formation and migration (Hall, 1998; Ridley et al., 1992), either uniformly within the gastrulating embryo (Fig. 4B-B″,F) or specifically within transplanted ppl progenitor cells (Fig. 4H-H″,K). Strikingly, we found that in both of these cases, ppl progenitor cells failed to undergo internalization (Fig. 4B-D,F; Movie 8). Moreover, ppl progenitors overexpressing DN-Rac did not show any preferentially localization of actin to their front ends *in vivo* (Fig. 4H-H″,J), and displayed reduced protrusion formation *in vitro* when cultured on fibronectin-coated substrates (Figs S2 and 3; Movie 9). Importantly, overexpression of DN-Rac did not affect differential TST-driven envelopment of ectoderm by mesoderm tissue in culture (Fig. S4), suggesting that DN-Rac does not strongly interfere with the differential TST that these tissues display. Collectively, these findings suggest that directed ppl progenitor cell migration plays a crucial role in progenitor cell internalization and segregation during gastrulation.

## DISCUSSION

Previous studies have shown that germ layer progenitor cell segregation in culture is driven by differences in TST among the forming germ layers, with ectoderm displaying higher TST than mesoderm and endoderm (Krieg et al., 2008; Maître et al., 2012; Schötz et al., 2008). Here, we show that this difference in germ layer TST crucially depends on the osmolarity of the surrounding fluid interface, and that within the gastrulating embryo under physiological osmolarity levels, this difference in TST diminishes. This argues against TST playing an instructive role in germ layer progenitor cell segregation during zebrafish gastrulation.

Osmolarity has previously been shown to affect cell interfacial tensions by altering hydrostatic cell pressure that in turn is balanced by cortex tension (Lang et al., 1998; Salbreux et al., 2012; Stewart et al., 2011). So far, studies on the interplay between medium osmolarity and hydrostatic cell pressure have mostly focused on cell responses to changes in medium osmolarity on timescales of seconds to minutes. By contrast, progenitor cell segregation both *in vitro* and *in vivo* occurs over a period of minutes to hours, and thus we recorded the response of progenitor cells to changes in medium/IF osmolarity on comparably long timescales. Consequently, the response of ectoderm and mesendoderm progenitors to changes in medium/IF osmolarity in our analysis describes the specific ability of those cell types in maintaining fluid homeostasis rather than their immediate response to changes in hydrostatic pressure. How, over such comparably long timescales, medium and/or IF osmolarity affects progenitor cell interfacial tensions is not yet clear, but the ability of progenitor cells to undergo regulated volume increase or decrease in response to osmotic swelling or shrinkage, and associated changes in the ionic composition of the cell cytoplasm are likely involved. A systematic analysis of how medium and/or IF osmolarity affects progenitor cell interfacial tensions, and how the acquisition of different cell fates by those progenitor cells modulates their response to IF and/or medium osmolarity will be needed to further explore how osmolarity functions in gastrulation movements.

We also show that instead of differential TST driving germ layer progenitor cell segregation, directed migration of mesendoderm cells from the outside to the inside of the germ ring margin is required for mesendoderm cell internalization during gastrulation. Why mesoderm cells polarize and migrate from the outside to the inside of the germ ring is still unclear, but one possibility is that the blastoderm displays an overall polarity along the radial axis of the embryo, and that this tissue polarity then triggers mesendoderm polarization and internalization. Supporting this assumption are previous findings that progenitor cells show a preferential localization of their microtubule organizing centers (MTOCs) along the radial axis of the blastoderm (Sepich et al., 2011) and our own observation of a graded distribution of IF accumulations from the outside to the inside of the germ ring (Fig. S5). How such polarized IF distribution is established within the blastoderm, and how it would trigger mesendoderm polarization is yet unknown. One possibility is that osmolarity-driven water influx over the EVL (Fukazawa et al., 2010; Kiener et al., 2008) creates a pressure gradient from the outside to the inside of the blastoderm, which leads to a graded distribution of IF along this axis (Fig. S5). As a result of this polarized IF distribution, mesendoderm progenitors might preferentially be in contact with IF closer to the germ ring outside, where more of it can be found, and this polarized IF interface might in turn trigger radial mesendoderm polarization. To test this assumption, techniques need to be developed that would allow direct analysis and manipulation of IF distribution within the developing embryo.

The role of differential TST in early development is still debated. Our CellFIT-3D-based analysis of cell interfacial tensions within the gastrulating embryo provides the first direct evidence that differential TST is not sufficient to explain germ layer progenitor cell segregation during zebrafish gastrulation. This does not argue against differential TST playing other important roles in early development, but clearly shows that complex morphogenetic processes, such as the formation and positioning of the different germ layers during gastrulation, depend on the interplay between different processes, including directed cell migration and polarization.

## MATERIALS AND METHODS

### Zebrafish handling

Zebrafish maintenance was carried out as described previously (Westerfield, 1993). Embryos were grown at 28-31°C in Danieau's embryo medium and staged as described previously (Kimmel et al., 1995). The following wild-type (WT), mutant and transgenic lines were used: (WT) TL; (mutant) maternal zygotic (MZ) *oep* (Gritsman et al., 1999); (transgenic) Tg*(dharma:eGFP)* (Ryu et al., 2001), Tg*(-4gsc:eGFP-Hsa.HRAS)*, Tg*(bAct:hRas-eGFP)* (Cooper et al., 2005), Tg*(bAct:myl12.1-eGFP)* and Tg*(bAct:myl12.1-mCherry)* (Maître et al., 2012); and Tg*(bAct:LifeAct-eGFP)* and Tg*(actb1:mCherry–utrCH)* (Behrndt et al., 2012).

### Embryo microinjections

Zebrafish embryos consisting of one germ layer progenitor cell type only were obtained by injection of one-cell stage embryos with either 100 pg *lefty1* mRNA (ectoderm) or 100 p*g ndr2*/*cyclops* mRNA plus 2* ng casanova morpholino* (*cas* MO; mesoderm) (Krieg et al., 2008). To visualize the plasma membrane and filamentous actin, 100 p*g lyn-TagBFP* or *lyn-Venus* (plasma membrane), and 50-100 p*g LifeAct-eGFP* (F-actin; Behrndt et al., 2012) were injected at the one-cell stage. To inhibit cell protrusion formation, one-cell stage embryos were injected with 400 pg *DN-Rac m*RNA. To distinguish between ectoderm and mesoderm populations in cell sorting and tissue envelopment experiments, embryos were additionally injected with Dextran-FITC or Dextran-tetra-methyl-rhodamin-dextran (TMR-dextran, LifeTechnologies) at the one-cell stage to label the cell cytoplasm. To visualize interstitial fluid (IF), 0.5-1.0 nL of 0.1% (w/v) TMR-dextran or Dextran-Cascade Blue (LifeTechnologies) were injected between deep cells of sphere-stage embryos (4 hpf) or tissue explants.

### Cell transplantations

Donor and host embryos were dechorionated with forceps and transferred into an agarose plate with Danieau's embryo medium. For the exogenous mesoderm internalization assay, two to five cells were taken from a mesoderm-induced donor embryo using a bevelled borosilicate needle with a 20 μm inner diameter attached to a syringe system, and transplanted directly below the surface cells close to the dorsal blastoderm margin of a dome stage (4.5 hpf) host embryo. For clonal analysis of actin and myosin II subcellular localization in progenitor cells during internalization, 20-50 cells from Tg(*bAct:myl12.1-eGFP*) or Tg(*bAct:LifeAct-eGFP*) donor embryos at sphere stage (4 hpf) were transplanted into the dorsal germ ring margin of a host embryo at the same stage.

### *In vitro* cell sorting/tissue envelopment assays

Zebrafish embryos were kept at 28-31°C until they were dissociated into single cells at sphere stage (4 hpf). Cell-sorting experiments were performed as described previously (Klopper et al., 2010), with the following modifications: micro-molds with a diameter of ±400 μm and a height of ±800 μm were made from a polydimethylsiloxane (PDMS) negative obtained from www.microtissues.com according to the supplier's guidelines. The different germ layer progenitor cell types were isolated and mixed by first removing the embryo animal poles of both mesoderm- and ectoderm-induced embryos (see above). For tissue envelopment assays, the animal poles of sphere-stage embryos (4 hpf) were cut into four equally sized pieces that were left to round up for 1 h at room temperature (22-25°C). One animal pole tissue piece from a MZ*oep* mutant embryo (ectoderm) was then co-cultured with a similarly sized piece from a mesoderm-induced embryo in a micro-well, and their envelopment behavior was recorded for at least 5 h at 28.5°C. For cell-sorting experiments, the same number of animal poles from ectoderm- and mesoderm-induced embryos were pooled in a tube and dissociated by gently tapping the tube. The resulting heterotypic ectoderm-mesoderm cell mixture was then seeded on micro-wells, and their sorting was recorded for at least 5 h in 3D over time at 28.5°C by acquisition of 4 μm spaced *z* stacks of the aggregate in two or three channels every 5 min, using a Leica SP5 confocal microscope equipped with a Leica 25×0.95NA dipping lens. All experiments were performed in CO_2_-independent DMEM/F12 medium, or water dilutions of it to lower medium osmolarity. For osmolarity rescue experiments, diluted medium was supplemented with mannitol (Sigma-Aldrich).

### *In vitro* cell protrusion assay

Glass-bottom dishes (MatTek) were coated with fibronectin by adding 50 μL of 200 μg/ml bovine fibronectin (Sigma), air dried at room temperature and overlaid with 50* *mg/mL BSA (Invitrogen) for 10 min. Mesoderm (prechordal plate) progenitor cells were isolated by first removing the animal poles of mesoderm-induced embryos (see above), and cutting them into smaller pieces with watchmaker forceps. Cell-clusters were seeded at 1×DMEM/F12 medium (Invitrogen) and left to adhere on fibronectin-coated glass substrates for 60-90 min prior to imaging. Imaging was performed on an inverted microscope (Axio Observer Z1 Zeiss) equipped with an automated TIRF/Epi-fluorescence system (Visitron Systems), with 488* *nm and 561* *nm laser lines. Images were acquired using a 20×objective (Zeiss) and an EMCCD camera (Evolve, Photometrix) with frame rates of 2 min and exposure times of 20-500 ms. Protrusion analysis was performed by segmenting out the seeded cells, from the images and comparing the perimeter length of the segmented area to the perimeter of an ellipsoid that was fitted to have the same area, and the same longest axes. An ellipsoid was chosen to compensate for unequal spreading or tissue stretching.

### IF osmolarity measurement

Donor and host embryos were dechorionated with forceps and transferred into an agarose plate containing Danieau's embryo medium. Animal poles were cut from the embryo at high-to-sphere stage (3-4 hpf), left to round up for 0.5 h at room temperature (22-25°C), and then cultured in Danieaus embryo medium at 28.5°C (Krens et al., 2011). After 2-4 h, when a clear fluid-filled cavity was formed at the explant interior, the IF was extracted and pooled from two to four explants per measurement using a bevelled borosilicate needle with a 10 μm inner diameter attached to a syringe system. The aqueous IF solution was transferred into a droplet of Cargille immersion type B mineral oil placed within a metal sample-holder plate of a nanoliter osmometer (Otago Osmometers). Osmolarity was determined by snap-freezing the IF droplet and recording the melting-temperature of the frozen droplet by visual inspection. Experiments were performed according to the suppliers' instructions, with the following adjustments: to reach sufficient cooling for freezing the sample, 40-60% ethanol-water solution was used as cooling fluid.

### Embryo imaging

Embryos were mounted in 0.7% low melting point agarose in Danieaus embryo medium. To record high-resolution time-lapse movies of cells and tissues deep within the embryo, a TriM Scope multi-photon microscope (LaVision BioTec) was used equipped with a multi-photon laser (Chameleon from Coherent) set to 830* *nm and an OPO laser set to 1100* *nm for exciting mCherry-labeled proteins. Image stacks of 70-150 μm with 2 μm *z* spacing were recorded in continuous mode, resulting in an image sampling rate of 4-6 min.

### Image analysis

Quantification of signal intensity and cell size was performed by analyzing images with Fiji (Schindelin et al., 2012). Intensity plots were generated by normalization to the average fluorescence intensity value and by scaling the front to the rear perimeter length. At least eight independent measurements were averaged and their mean values were displayed as polar plots with standard deviation. The ratios between the cell-front and cell-rear intensities were calculated using Matlab (Mathworks) and Excel (Microsoft). Quantification of relative fluid occupation was plotted as a percentage of fluid occupation over the total distance from the epiblast surface by normalizing to the 256 gray values of the 8-bit image. The obtained values were averaged over 10 μm bins from the epiblast surface (0 μm) to its inside (50 μm). Cell tracking, orthogonal views and 3D-renderings were generated using Imaris version 7.3 (Bitplane). The distance to the enveloping layer (EVL) was calculated from the 3D coordinates of manually generated cell tracks for mesoderm, ectoderm and EVL cells. Statistical data analysis was performed using the GraphPad Prism 5 software.

### Single cell force spectroscopy

Cell-cortex tension measurements were performed as described previously (Krieg et al., 2008) with the following adjustments: the animal poles were mechanically removed in E3 medium from either ectoderm (MZoep) or mesoderm-induced embryos using watchmakers forceps and directly transferred into a 3.5 cm petri dish containing 4 ml 0.8×DMEM/F12 (240 mOsm/L). Five animal poles each were then transferred to either 1×DMEM/F12 (300 mOsm/L) or 0.63×DMEM/F12 (190 mOsm/L). The animal poles were mechanically dissociated and individual cells were seeded on a glass substrate. Cells were probed with colloidal force probes, which were prepared by attaching a glass bead (5 μm diameter, Kisker Biotech) to a cantilever (Veeco MLCT). To prevent non-specific adhesion of the cantilever/bead to the cells, the modified cantilevers were incubated with heat-inactivated fetal calf serum for at least 1 h at room temperature (FCS, Invitrogen) prior to the measurements. Force-distance curves were acquired using 500 pN contact force and 1 μm s^–1^ approach/retract velocity and indentation (δ) was calculated from the tip displacement. Up to three curves with at least 10 s waiting time between successive curves were taken per cell to prevent any history effect. The liquid droplet model was applied to extract the cell-cortex tension as described previously (Krieg et al., 2008), with the following adjustments: to determine cell-cortex tension we used a force versus indentation line-fit between a 200* *nm and 300* *nm indentation range.

### CellFIT-3D analysis

To obtain estimates of the relative edge tensions, the angles at triple junctions, such as those between a mesoderm cell, ectoderm cell and the medium, were digitized using custom software as described in the supplementary Materials and Methods. Angles along particular edges were digitized in multiple images within the stack in order to obtain the true angles of the cell membranes with the edge. Force-balance equations were written for each digitized triple junction, and least-squares solutions were found for all such equations. The solutions to these equations provided the relative strengths of the tensions along each edge type.

### Finite element simulations

The simulations were carried out using the finite element formulation described previously (Brodland and Chen, 2000), which assumes that cell-cell and cell-medium interfaces carry net interface-specific tensions and the cytoplasm and other contents of the cells generate an effective viscosity that can be described by an orthogonal system of dashpots. In cases where cortical tensions varied within a particular cell, the tension applicable to any particular edge was based on the location of its midpoint. All calculations were carried out using an updated Lagrangian approach and the simulations were run until motion stopped. For all simulations, effective viscosity in ectoderm and mesoderm cells was assumed to be the same, and variation of this parameter did not change the principal outcome of cell segregation.
